# Cost-effectiveness of metacognitive therapy for cardiac rehabilitation participants with symptoms of anxiety and/or depression: analysis of a randomised controlled trial

**DOI:** 10.1136/bmjopen-2024-087414

**Published:** 2024-12-20

**Authors:** Gemma E Shields, Elizabeth Camacho, Linda M Davies, Patrick Joseph Doherty, David Reeves, Lora Capobianco, Anthony Heagerty, Calvin Heal, Deborah Buck, Adrian Wells

**Affiliations:** 1 Manchester Centre for Health Economics, University of Manchester, Manchester, UK; 2 Department of Health Sciences, University of York, York, UK; 3 Centre for Primary Care, University of Manchester, Manchester, UK; 4 Greater Manchester Mental Health NHS Foundation Trust, Manchester, UK; 5 Division of Psychology & Mental Health, University of Manchester, Manchester, UK; 6 Division of Cardiovascular Sciences, University of Manchester, Manchester, UK; 7 Centre for Biostatistics, University of Manchester, Manchester, UK

**Keywords:** REHABILITATION MEDICINE, CARDIOLOGY, HEALTH ECONOMICS, Anxiety disorders, Depression & mood disorders

## Abstract

**Abstract:**

**Objectives:**

The burden of cardiovascular disease (CVD) is increasing. Cardiac rehabilitation (CR) is a complex intervention offered to patients with CVD, following a heart event, diagnosis or intervention, and it aims to reduce mortality and morbidity. The objective of this within-trial economic evaluation was to compare the cost-effectiveness of metacognitive therapy (MCT) plus usual care (UC) to UC, from a health and social care perspective in the UK.

**Methods:**

A multicentre, single-blind, randomised controlled trial (ISRCTN74643496) was conducted in the UK involving 332 patients with CR with elevated symptoms of anxiety and/or depression and compared group-based MCT with UC. The primary outcome of the cost-effectiveness analysis was quality-adjusted life-years (QALYs). The time horizon of the primary analysis was a 12-month follow-up. Missing data were imputed using multiple imputation. Uncertainty was explored by probabilistic bootstrapping. Sensitivity analyses tested the impact of the study design and assumptions on the incremental cost-effectiveness ratio.

**Results:**

In the primary cost-effectiveness analysis, MCT intervention was dominant, with a cost-saving (net cost −£219; 95% CI −£1446, £1007) and QALY gains (net QALY 0.015; 95% CI −0.015, 0.045). However, there is a high level of uncertainty in the estimates. At a threshold of £30 000 per QALY, MCT intervention of around 76% was likely to be cost-effective.

**Conclusions:**

Results suggest that intervention may be cost-saving and health-increasing; however, findings are uncertain and subject to limitations. Further research should aim to reduce the uncertainty in the findings (eg, with larger sample sizes) and explore potential longer-term economic benefits associated with MCT in this setting.

**STRENGTHS AND LIMITATIONS OF THIS STUDY**

- The current study is the first economic evaluation of metacognitive therapy (for any population group) and uses prospectively collected data from a high-quality randomised controlled trial.
- The level of missing data increases uncertainty in the study results; however, this is investigated by using and comparing a multiple imputed approach with a complete case analysis.
- The study was constrained by the design of the clinical trial; in particular, the limited follow-up may not capture all economic impacts.

## Background

 The burden of cardiovascular disease (CVD) is increasing globally; prevalent cases have nearly doubled from 1990 to 2019 (from 271 million to 523 million).^1^ Deaths from CVD grew globally to 18.6 million in 2019, representing the most common cause of death across European Society of Cardiology member countries.^1 2^ The morbidity impact of CVD is considerable with an estimated 34.4 million years lived with disability in 2019.^1^ The economic burden associated with CVD is substantial; healthcare costs related to CVD are estimated to be £7.4 billion and costs to the wider economy are an estimated £15.8 billion per year in the UK.^3^ Pre-COVID-19 CVD estimates are highly likely to underestimate future burden. For example, a recent British Heart Foundation analysis revealed around 100 000 CVD-related excess deaths in England since the beginning of the pandemic.^4^

Cardiac rehabilitation (CR) is a complex intervention offered to patients with CVD following a heart event, diagnosis or intervention (criteria vary by setting), which commonly includes education, physical activities/exercise and psychological intervention components.^5^ Benefits of CR include reductions in cardiac event reoccurrence, mortality risk and rehospitalisation.^6^,^9^ Furthermore, CR has generally been demonstrated to be cost-effective.^10^ Before the COVID-19 pandemic, in England, Wales and Northern Ireland, around 80 000 people initiated CR annually (2019 data).^11^ During the pandemic, there were changes to CR delivery (eg, staff redeployment and lockdowns).^12^ However, the need for CR services remains high.^13^

The British Association for Cardiovascular Prevention and Rehabilitation (BACPR) recommends that CR should incorporate key components, including lifestyle risk factor management, long-term strategies, psychosocial health, health behaviour change, and education and medical risk management.^14^ One of the key components is incorporating interventions to promote psychosocial health, though the precise requirements of such an intervention are not standardised and they vary by CR programme. A common intervention includes stress management, containing a mixture of techniques that often varies by site but can include relaxation, thought challenging and coping techniques, although a review concluded that this may only have a small impact on anxiety and depression, and the evidence base is limited by the quality of the evidence.^15^ Furthermore, a review of the cost-effectiveness of CR found a paucity of evidence to support psychological interventions.^10^

In the recent MCT-PATHWAY research programme, the mental-health benefits associated with adding metacognitive therapy (MCT) to CR were evaluated.^16 17^ Unlike existing psychological treatment approaches in CR, MCT is based on a specific and highly structured treatment manual for treating anxiety and depression. MCT is grounded in an evidence-based theory of psychological disorder that proposes that a particular thinking pattern, dominated by worry, rumination and threat monitoring, maintains emotional distress symptoms.^18^,^20^ Such a thinking style results from distorted metacognitive beliefs (eg, ‘I have no control over my health worries’) that prevent individuals from effectively regulating negative thought patterns and reducing distress. In patients undergoing CR, thoughts can be dominated by worries (eg, concern about having another cardiac event), resulting in repetitive negative thinking^21 22^ which in the presence of negative metacognitive beliefs is difficult to regulate. MCT is a treatment that is designed to modify such underlying metacognitions, and it improves the choice and use of strategies for regulating thinking.^18^ Subsequently, MCT has the potential to improve health in the CR population as it focuses on reducing negative repetitive thinking, and as a further benefit, unlike current therapies, it allows patients to tackle this without in-depth analysis of thought content, which may be challenging for patients with CR as their concerns are often realistic.

The MCT-PATHWAY programme involved a multicentre, single-blind, randomised controlled trial that evaluated the effectiveness and cost-effectiveness of group-based MCT for patients with CR with elevated anxiety and/or depressive symptoms. Details of the trial methods and results are published elsewhere.^16 17^ The trial found that Group MCT plus usual CR was associated with moderate to large reductions in anxiety and depression, unhelpful metacognitions and repetitive negative thinking in comparison with CR alone.^17^ The programme results are also available in the National Institute of Health Research (NIHR) journal library.^23^ These results add to the growing evidence of the effectiveness of MCT for anxiety and depression.^24^

To date, there have been no cost-effectiveness studies of MCT, and a review of cost-effectiveness studies in CR identified only two studies evaluating other psychological support in CR, with mixed results and limited generalisability to UK practice.^10^ Cost-effectiveness evidence is required to support decision-makers by providing information on the trade-off between costs and clinical impact of an intervention to allow for an assessment of whether the proposed intervention offers value for money.

Given the potential beneficial effects associated with MCT on anxiety and depression in CR and the lack of existing cost-effectiveness data, we embedded a preliminary economic evaluation into the MCT-PATHWAY trial. The aim of this study was to assess whether the addition of MCT to CR for patients with elevated symptoms of anxiety and/or depression is potentially cost-effective compared with usual care (UC).

## Methods

A within-trial economic evaluation compared the cost-effectiveness of MCT plus UC to UC alone, from a health and social care perspective in the UK.

### Protocol

The protocol for the economic evaluation is available.^25^

### Design, setting and participants

Full details of the trial methods and results, as well as wider work from the funded programme of research, are available elsewhere.^16 17 21 22 26^ The full programme results (including an overview of this economic evaluation) are available in the NIHR journal library publication.^23^ In brief, the group-MCT trial recruited 332 patients with CR from five NHS sites in the North-West of England. Participants were over 18 years old and met the Department of Health or BACPR CR eligibility criteria, as well as having a competent level of English language skills. Patients referred for CR are sent a National Audit of Cardiac Rehabilitation (NACR) assessment pack, which includes the Hospital Anxiety and Depression Scale (HADS) and, to be eligible for the trial, patients had to score 8 or above on either the anxiety or depression subscale of HADS. Exclusion criteria included cognitive impairment, life expectancy below 12 months, suicidality, active psychotic disorders, drug or alcohol abuse, recent initiation of antidepressant or anxiolytic medications and concurrent psychological intervention.

Participants were randomly allocated (balanced by sex and HADS score within sites) to receive either group-MCT plus CR or CR alone. Usual CR included home and centre-based group interventions such as exercise sessions, educational seminars and stress management (eg, relaxation), and the precise contents of CR varied by site. Group MCT consisted of six weekly sessions with a duration of 60 to 90 min and was delivered by two trained CR professionals at each site.

The primary outcome in the trial was HADS total score, with secondary outcomes capturing posttraumatic stress symptoms, metacognitions, mechanism variables and outcomes required for economic evaluation. Participants completed a baseline assessment prior to randomisation (distinct from the initial NACR assessment) and further assessments at the 4-month and 12-month follow-up. While the clinical trial gave primacy to outcome scores at the 4-month follow-up, the present economic evaluation focuses on longer-term outcomes at the 12-month follow-up to incorporate sufficient time for any impact of MCT on service use and health benefit.

### Health-related quality of life and quality-adjusted life-years

The measure of health benefit used was quality-adjusted life-years (QALYs) over 12 months, estimated using EQ-5D-5L which was collected at baseline, 4-month and 12-month follow-up. EQ-5D is a generic measure of health status, validated in the population, and recommended for use in economic evaluation by NICE.^27 28^ In line with NICE recommendations at the time of the analysis, the crosswalk algorithm was used to estimate utility values from EQ-5D.^29^ Total QALYs were calculated using an area under the curve approach, accounting for utility at each assessment and duration of time between assessments.

### Resource use and costs

Data on health and social care use were collected using an economic patient questionnaire adapted from other trials.^30^ This captured inpatient, outpatient, day case, accident and emergency, primary, community and social care use. Unit costs of NHS and social care services (online supplemental file 1) were derived from national average unit cost data, and the price year was 2019. Staff time to deliver MCT and CR attendance was collected by the research team. The cost of CR sessions (both education and exercise) was £48 per participant per session.^31^ In the primary analysis, MCT costs included staff time for preparation and delivery, and the costs were associated with providing a manual and CD. The cost of manual and CD was negligible (£3.55). Staff costs were estimated using the mean of a range of staff at Band 6 and Band 7, including community nurses, hospital-based physiotherapists and occupational therapists.^32^ Two healthcare practitioners were paid to deliver sessions, with 2 hours assumed to cover preparation and delivery. The cost per participant was calculated using the average group size from the trial. This resulted in the mean cost of £54 per MCT session per participant which was multiplied by the number of sessions attended.

### Analysis

The within-trial analysis used intention-to-treat and estimated total costs and QALYs for the trial follow-up. The time horizon of the within-trial primary analysis was 12 months to incorporate sufficient time for any impact of MCT on service use and health status.

Multiple imputation was used to impute values missing at follow-up. Costs were imputed by service category and utility by EQ-5D domain to use all available data. Regression analysis was used to estimate net costs (generalised linear model) and net QALYs (linear regression model), adjusted for participant characteristics (baseline covariates). Covariates for costs and QALYs included age, gender, hospital site, baseline HADS score, medication for depression or anxiety, BMI, smoking status, alcohol units consumed per month and number of comorbidities.

The primary measure of interest was the incremental cost-effectiveness ratio (ICER), calculated as:

$$ICER=\;\frac{Cost_{intervention}-Cost_{comparator}}{QALYs_{intervention}-\;QALYs_{comparator}}$$

Net costs and net QALYs estimates were bootstrapped to generate 10 000 pairs of costs and QALYs to inform the probability of cost-effectiveness. Each of the net QALY estimates from bootstrap simulation results were revalued by multiplying it by willingness-to-pay thresholds (WTPT). Net monetary benefit statistics were produced for each pair of simulated net costs and net benefits. The monetary value of simulated QALYs varied from £0 to £30 000 to reflect a range of hypothetical WTPT.

Key sensitivity analyses, specified a priori, were used to test the impact of the study design on the results of the cost-effectiveness analysis (table 1).

**Table 1:** Sensitivity analysis rationale

Sensitivity analysis	Rationale
Complete case analysis	Multiple imputation is increasingly at risk of bias and imprecision as the amount of missing data increases. However, complete case analysis may be biased as the sub-sample may not be representative of all trial participants. Therefore, the primary analysis using multiple imputation will be compared with a complete case analysis to assess whether results indicate similar conclusions.
Alternative measures of benefit	EQ-5D-5L is a general measure of health, recommended for use in economic evaluation to calculate a QALY. However, there is debate about whether this is sensitive to clinically relevant changes in mental health. Therefore, a sensitivity analysis was conducted using the primary clinical outcome measure (HADS). However, it is important to note that there is no commonly accepted WTPT for this measure.
Anxiety and/or depression at baseline	Trial eligibility criteria included a HADS anxiety or depression score of ≥8 at the NACR assessment. However, when the baseline measures were taken, some participants no longer met this criteria. An analysis explored the impact of restricting the sample to participants meeting the HADS criteria at baseline assessment.
Treatment received rather than intention-to-treat analysis	The primary analysis was intention-to-treat; however, not all patients assigned to the MCT intervention attended any sessions. Therefore, an exploratory analysis was conducted using only the participants in the intervention arm who had attended one or more MCT sessions.
Unit costs of intervention	Two sensitivity analyses varied the costs of MCT. One assumed lower costs due to a larger average group size, considered likely if MCT was implemented in CR since this reduces the cost per participant. Another sensitivity analysis explored the inclusion of training and supervision costs related to MCT intervention, which increases intervention costs.
Time horizon	A further sensitivity analysis focused on the 4-month follow-up (the primary follow-up of the trial) to assess the impact of different follow-up periods on cost-effectiveness results.

CRcardiac rehabilitationHADSHospital Anxiety and Depression ScaleMCTmetacognitive therapyNACRNational Audit of Cardiac RehabilitationQALYquality-adjusted life-yearWTPTwillingness-to-pay thresholds

Data manipulation and analysis were conducted using SPSS V.25 and Stata V.14. Further details on the methods can be found in the published protocol.^25^

### Deviations from protocol

The original protocol detailed several additional potential sensitivity analyses; however, these analyses were not conducted due to the sample size and completeness of data within the sample. For example, we had considered it possible to conduct subgroup analyses; however, as the sample size within specific subgroups is limited, this would not be robust and would risk false negatives due to a lack of statistical power.^33^ Originally, it was planned that a de novo economic model would be constructed with the aims of exploring (1) the cost-effectiveness of MCT over a longer time horizon and (2) the cost-effectiveness of MCT in different populations and settings. A preliminary model design was drafted. However, during the model design, discussions highlighted that without additional evidence becoming available, the economic model would not be robust. In particular, high-quality data generalisable to the UK are needed to support the rates of relapse and remission of depression and/or anxiety symptoms specific to the CR population, mortality rates in the CR population with and without anxiety and/or depression, evidence to support long-term effectiveness of MCT for this population and data on interactions between mental and physical health in this population.

### Patient and public involvement

The PATHWAY Group MCT Patient and Public Involvement (PPI) has been reported in a separate publication.^34^ Specific to the economic evaluation, the PPI group reviewed and commented on all trial questionnaires (including the economic patient questionnaire) before finalisation.

## Results

### Participants and completion

Trial participants’ baseline characteristics are reported in the trial publication, as noted in the trial publication groups were well-balanced on measured characteristics.^17^

Cost and QALY data were complete at all three time points for 179 participants (54%; 91 control, 88 intervention). A total of 339 participants had complete EQ-5D data at baseline, 260 (78%) at the 4-month follow-up and 245 (74%) at the 12-month follow-up. At baseline, 262 (79%) participants had sufficient data from the service use questionnaire to estimate baseline costs, while at the 4-month follow-up it was 203 (61%) and at the 12-month follow-up it was 211 (64%).

### Costs and health status

Online supplemental file 1 includes the mean utility value at each assessment. Responses to the EQ-5D confirm the impact of coexisting physical and mental health difficulties in the samples, with the majority of domains greatly affected.

A cost breakdown by category of service use and by arm is also included in online supplemental file 1. Note that a high level of variation is common with costing data and is demonstrated here. Inpatient costs are high at baseline as would be expected based on the trial inclusion criteria, that is, inpatient costs at baseline reflect that participants had a recent cardiac event and thus would have been probably hospitalised. A similar trend is seen with accident and emergency costs. Costs related to CR attendance are not significantly different across groups, suggesting that MCT intervention did not impact the decision to attend the usual CR offering.

### Cost-effectiveness analysis

Table 2 reports the net costs and QALYs estimated by the bootstrap simulation of the multiple imputation data, adjusted for any differences between the groups in baseline characteristics (covariates).

**Table 2:** Net costs and QALYs, and probability MCT intervention is cost-effective (bootstrapped and adjusted for baseline covariates)

Analysis*	Net cost (95% CI)†	Net QALY (95% CI)	ICER (£ per QALY)†	Probability MCT is cost-effective compared to usual care at different WTPTs		
				£0 per QALY	£15 000 per QALY	£30 000 per QALY
Primary (n=332)	−£219 (−£1446; £1007)	0.015 (−0.015; 0.045)	Dominant	59%	70%	76%
Sensitivity analysis						
Complete case (n=179)	−£1 (−£1387; £1385)	0.035 (−0.004; 0.074)	Dominant	43%	69%	83%
Participants with anxiety and/or depression confirmed by HADS at baseline (n=284)	£75 (−£1090; £1241)	0.013 (−0.020; 0.045)	£5901	39%	51%	60%
Treatment received rather than intention-to-treat analysis (n=292)‡	£133 (−£1166; £1432)	0.015 (−0.018; 0.049)	£8618	35%	48%	58%
MCT costs (inclusive of training and supervision) (n=332)	−£9 (−£1225; £1207)	0.015 (−0.015; 0.045)	Dominant	43%	58%	67%
MCT costs (larger group size) (n=332)	−£356 (−£1604; £891)	0.015 (−0.015; 0.045)	Dominant	68%	77%	82%
Alternative measure of benefit (HADS) (n=332)§	−£219 (−£1446; £1007)	−1.999 (−3.537; −0.61)	Dominant	59%‡	99%‡	99%‡
Time horizon (4-month follow-up) (n=332)	−£175 (−£832; £482)	0.005 (−0.008; 0.018)	Dominant	60%	69%	74%

* Unless stated otherwise, net costs and health benefits adjusted for baseline covariates using imputed data, bootstrapped 10 000 times.

† Costs given in £’s, 2019.

‡ Note this does not reflect a clinically significant dose (≥4 sessions), rather whether participants attended any intervention sessions (≥1 session/s).

§ There is no accepted threshold or range of threshold for a unit change in HADS. It is left to decision-makers to consider how much they would be prepared to pay for a specific health gain.

HADSHospital Anxiety and Depression ScaleICERincremental cost-effectiveness ratioMCTmetacognitive therapyQALYquality-adjusted life-yearWTPTwillingness-to-pay thresholds

In the primary cost-effectiveness analysis at the 12-month follow-up, the MCT intervention appears dominant, meaning it is both cost-saving (net cost -£219; 95% CI -£1446, £1007) and health-increasing (net QALY 0.015; 95% CI −0.015, 0.045). However, the CIs are wide and overlap zero, indicating a high level of variability in the data and uncertainty in the estimates. The primary analysis found that at a threshold of £30 000 per QALY, the MCT intervention is around 76% likely to be cost-effective, again reflecting uncertainty. Similarly, in the majority of sensitivity analyses, the MCT intervention is dominant (cost-saving and health-increasing); however, there is high uncertainty as indicated by the CIs.

**Figure 1:**
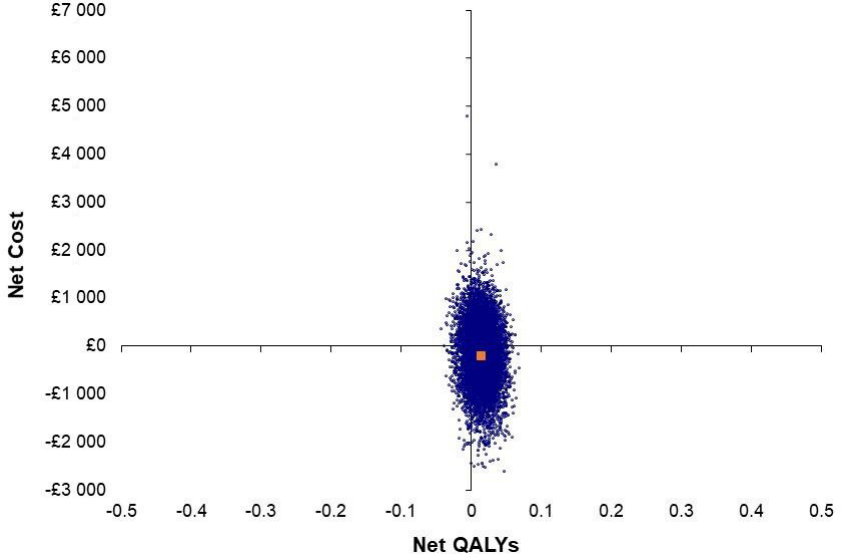
Cost-effectiveness plane (placeholder). QALYs, quality-adjusted life-years.

**Figure 2:**
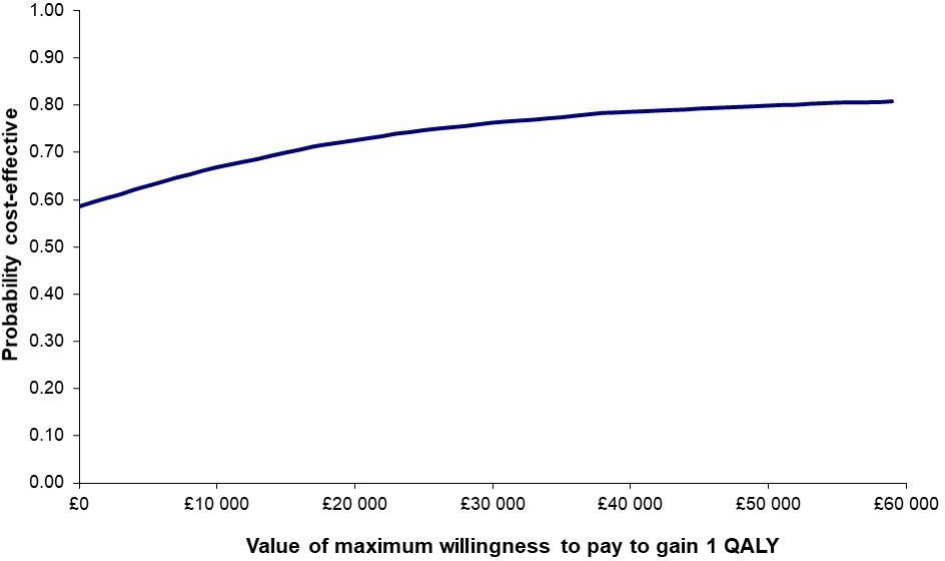
Cost-effectiveness acceptability curve (placeholder). QALYs, quality-adjusted life-years.

Figure 1 displays the cost-effectiveness plane for the primary analysis, which shows the distribution of net costs and QALYs. The uncertainty in the analysis is demonstrated as the net cost/QALY pairs are spread across each of the four quadrants. Figure 2 shows the cost-effectiveness acceptability curve for MCT intervention, which shows that as the willingness to pay per QALY increases, so does the likelihood of MCT intervention being cost-effective.

Sensitivity analysis (table 2) demonstrated that the results at the 4-month follow-up were similar to the primary analysis, and the complete case analysis or the use of different assumptions around the cost of MCT did not affect conclusions. In these sensitivity analyses, MCT remained dominant but with CIs wide and overlapping zero demonstrating considerable uncertainty. In two sensitivity analyses, MCT was associated with a net cost increase, though again this was not statistically significant.

## Discussion

The primary cost-effectiveness analysis and the majority of sensitivity analysis indicate that MCT intervention may be cost-saving and health-increasing; however, the wide CIs that overlap zero indicate a high level of variability and uncertainty in the estimates. In the primary analysis, the probability of cost-effectiveness ranges from 59% at a threshold of £0 per QALY to 76% at a threshold of £30 000 per QALY.

Regarding the sensitivity analysis, a significant reduction in the HADS was demonstrated. While the decrease in HADS score is significant, the net costs are not significant. In the clinical effectiveness study, more participants in the MCT arm experienced a reliable improvement in their HADS score (21% in CR compared with 33% in MCT+CR), and fewer experienced a psychological deterioration (15% in CR compared with 4% in MCT+CR).^17^ It could be hypothesised that if sustained over longer time horizons, a more significant QALY gain could be seen (resulting from both improved mental and physical health as there are interactions between the two, especially in CR). Likewise, in the longer term, if the MCT intervention was able to affect repeat cardiac events (as some evidence suggests that symptoms of anxiety and/or depression are predictors of cardiac events) there could be substantial reductions in hospitalisation costs.^35^

The results at the 4-month follow-up are very similar to the primary analysis (12-month follow-up). The complete case analysis does not affect conclusions, and the uncertainty remains. As would be expected, different assumptions around the cost of MCT affect the probability of cost-effectiveness. A larger group size, which could be realistic given CR group sizes, reduces cost and increases the probability of cost-effectiveness, whereas the inclusion of training and supervision costs has the opposite effect. Outside of a trial setting, the level of training (and supervision) and the delivery of MCT may vary. These sensitivity analyses highlight the importance of considering these factors. The current CR offering for psychological support includes stress management techniques, which have a limited evidence base.^15^ In reality, with a focus on providing evidence-based practice, MCT may replace this current offering and subsequently the cost impact may be negligible or zero as the staff time to deliver previous offerings is simply shifted to providing MCT.

In two of the sensitivity analyses, MCT was associated with a net cost increase, though this was not statistically significant. Both of these analyses restricted the participant sample. The first analysis focused on those who met the HADS cut-off for depression and/or anxiety at baseline, excluding those who no longer met the criteria (ie, between screening and baseline the participant had a reduction in HADS score). The second analysis restricted the MCT arm to the participants assigned to the intervention who attended one or more sessions of MCT. While the ICERs estimated for these analyses were under commonly discussed thresholds, uncertainty remains. These explorative analyses highlight the need to consider how the implementation of MCT in CR will impact cost-effectiveness. For example, a substantial wait time for therapy will have a knock-on effect on cost-effectiveness.

Prior reviews identified sparse evidence for the cost-effectiveness of psychological intervention within CR and results were mixed.^1036^,^38^ Two existing studies focus on psychological therapy in CR; one study of a home-based cognitive-behavioural therapy found it to be cost-effective in the majority of cases compared with UC (67%), and the other focused on learning and coping education strategies which was cost-effective only 29% of the time.^39 40^ To the best of authors’ knowledge, as well as expanding the evidence base for psychological therapy in CR, the current study is the first economic evaluation of MCT (for any population group).

Another component of the wider PATHWAY study looked at preferences for psychological therapy delivery in CR.^34^ The discrete choice experiment (DCE) indicated that among the participants recruited, they would be more likely to opt-in to therapy (vs opting out) within CR. The results of the DCE suggest that adapting the offering to preferences may have cost implications. For example, the general population sample favoured individual therapy which would be more costly to deliver. Additionally, preference heterogeneity is an issue which may prevent a 'one-size-fits-all' approach to psychological therapy in CR, especially if focused on uptake. Important inequalities in uptake exist in CR, with lower uptake in more deprived areas, for minority ethnicities and for single and older people.^41 42^ Furthermore, practical barriers exist, such as financial costs, travel time and the need to take time off work/other commitments to attend.^43^ Uptake was not investigated in this study, but changes in CR design may affect uptake. For example, people may be more inclined to attend CR if there is a suitable psychological option. It has been noted that completing CR is challenging while experiencing psychological distress.^44^ A recently published analysis indicated that increasing uptake in CR can have a high justifiable expenditure (eg, due to offsets in hospitalisation costs).^45^ Investigation into whether MCT can positively affect uptake/attendance at CR and subsequent cost-effectiveness is needed. Furthermore, research into how MCT could avoid or overcome the current barriers to CR attendance would be beneficial.

During the COVID-19 pandemic, there was a shift towards home-based CR options.^12 13^ A separate pilot DCE investigated preferences delivery which indicated the participants tended to favour home-based psychological therapy in CR.^46^ However, qualitative interviews of patients attending group-MCT in the PATHWAY trial indicated that patients valued hearing the experiences of other patients in the group.^21 22^ Home-based MCT has been shown to be effective in reducing symptoms of anxiety and depression in the population receiving CR.^47^ Furthermore, home-based CR options have been demonstrated to be effective and cost-effective.^48^,^51^ Research is needed to compare the effectiveness and cost-effectiveness of clinic-delivered MCT versus home-based MCT in CR, and this is likely to consider patient preferences and how this will affect uptake/attendance.

The study used EQ-5D, recommended by NICE.^28 52^ The Recovering Quality of Life (ReQoL) measure is now available and is a generic self-report measure for use with people experiencing mental health concerns.^53^ In comparison with EQ-5D, ReQoL has more focus on mental health and quality of life, and it also allows for the estimation of utilities for use in economic evaluation. Subsequently, in future research, the exploration of different measures is recommended, as the EQ-5D cannot reflect all aspects of health for all diseases and patients. This should be considered when interpreting the results of the current economic evaluation, as the analysis with the mental health-focused outcome (HADS) had more favourable results.

The economic evaluation shared the strengths and limitations of the trial, reported elsewhere.^17^ Key strengths of the economic evaluation include the prospective collection of economic data alongside a robust randomized-controlled trial, a comprehensive investigation into uncertainty and a range of sensitivity analyses to explore the impact of assumptions related to methods/design. A key limitation of the economic evaluation is specifically related to uncertainty. While the trial achieved a high rate of follow-up at the primary time horizon (4 months), there was a relatively high level of missing data for costs and EQ-5D at the final follow-up (12 months). Overall, 54% of participants had complete cost and utility data at both baseline and follow-up. Data were imputed by category of cost and EQ-5D domain to make the best use of all available data. Furthermore, a complete case analysis was conducted for comparison. However, given the level of missing data, results should be interpreted with caution as higher levels of missing data reduce the robustness of imputation. The level of missing data is similar to other trials that have collected self-report data using a similar questionnaire in mental health populations.^54^,^56^ The sample size and missing data limited the potential for subgroup analyses. Health and social care service use was self-reported by trial participants. While this is a valid approach to data collection, especially in the UK where access to electronic data is associated with significant hurdles in terms of time and budget, it is open to recall bias and missing data.^57^ Service use and subsequently cost data often vary and the sample size of the study and data completeness limit conclusions. Furthermore, unit costs, especially those related to cardiac inpatient admissions, can be substantial and variable. Further research should investigate how the addition of psychological therapy impacts the categories of service use and the interactions between these categories to more robustly determine how the intervention may affect net costs across health and social care. Results may not be generalisable to other settings; cardiac services, including the type of CR offered (eg, face-to-face or home-based) and exact design and delivery of components (eg, exercise components), and populations vary by area.^40 41^ The NIHR is providing funding to examine the roll-out of MCT in six CR services across England (the BEACONS project). This may help supplement some of the evidence requirements to more thoroughly investigate the possible cost-effectiveness of MCT in CR. While there are some favourable results, more robust data are needed to make stronger conclusions around cost-effectiveness. It should be noted that the clinical findings for Group MCT are positive, with more moderate to large reductions in mental health symptoms (eg, anxiety and depression) when compared with CR alone.^17^

In conclusion, in the primary analysis using EQ-5D, MCT was dominant (cost-saving and health-increasing), though not statistically significant. However, results using an alternative measure of benefit (HADS) were significant. The analysis was subject to limitations, in particular the sample size and level of missing data. Further research should aim to reduce the uncertainty (eg, with larger sample sizes). Given the reduction of symptoms of anxiety and depression, there is a need to explore potential longer-term economic benefits associated with MCT in this setting and when assessed using more mental health-focused measures of health benefit.

## supplementary material

10.1136/bmjopen-2024-087414 online supplemental file 1

## Data Availability

Data are available upon reasonable request.
