# Supplementary material for: Cost-effectiveness of metacognitive therapy for cardiac rehabilitation participants with symptoms of anxiety and/or depression: analysis of a randomised controlled trial
Source: BMJ Open. 2024 Dec 20;14(12):e087414. doi: 10.1136/bmjopen-2024-087414 (PMC11667381; doi:10.1136/bmjopen-2024-087414)
Supplement: online supplemental file 1 [file bmjopen-14-12-s001.docx]

# A cost-effectiveness analysis of metacognitive therapy for cardiac rehabilitation participants with symptoms of anxiety and/or depression

Contact author: gemma.shields@manchester.ac.uk

# Supplementary Table 1: Unit costs

| **Service type** | **Unit cost** | **Reference (unit measure)** |
| --- | --- | --- |
| **Inpatient stays^1^** | | |
| Angiogram | £760 | NHS reference costs 2017/2018 updated to 2019 prices (per day) |
| Angioplasty elective | £1,819 | NHS reference costs 2017/2018 updated to 2019 prices (per day) |
| Angioplasty | £1,086 | NHS reference costs 2017/2018 updated to 2019 prices (per day) |
| Blood disorder non-elective | £495 | NHS reference costs 2017/2018 updated to 2019 prices (per day) |
| Bowel cancer elective | £1,480 | NHS reference costs 2017/2018 updated to 2019 prices (per day) |
| Breast surgery elective | £2,835 | NHS reference costs 2017/2018 updated to 2019 prices (per day) |
| Broken shoulder non-elective | £523 | NHS reference costs 2017/2018 updated to 2019 prices (per day) |
| Bypass elective | £2,299 | NHS reference costs 2017/2018 updated to 2019 prices (per day) |
| Bypass | £1,853 | NHS reference costs 2017/2018 updated to 2019 prices (per day) |
| Cardiology elective | £1,116 | NHS reference costs 2017/2018 updated to 2019 prices (per day) |
| Cardiology non-elective | £632 | NHS reference costs 2017/2018 updated to 2019 prices (per day) |
| Catheter procedures | £760 | NHS reference costs 2017/2018 updated to 2019 prices (per day) |
| Coronary elective | £2,261 | NHS reference costs 2017/2018 updated to 2019 prices (per day) |
| Coronary non-elective | £1,199 | NHS reference costs 2017/2018 updated to 2019 prices (per day) |
| Gastroenterology elective | £3,207 | NHS reference costs 2017/2018 updated to 2019 prices (per day) |
| General admission elective | £1,480 | NHS reference costs 2017/2018 updated to 2019 prices (per day) |
| General admission non-elective | £523 | NHS reference costs 2017/2018 updated to 2019 prices (per day) |
| ICU | £1,501 | NHS reference costs 2017/2018 updated to 2019 prices (per day) |
| Kidney non-elective | £461 | NHS reference costs 2017/2018 updated to 2019 prices (per day) |
| Neurology non-elective | £1,038 | NHS reference costs 2017/2018 updated to 2019 prices (per day) |
| Respiratory infection elective | £601 | NHS reference costs 2017/2018 updated to 2019 prices (per day) |
| Shoulder op elective | £2,871 | NHS reference costs 2017/2018 updated to 2019 prices (per day) |
| Stroke non-elective | £466 | NHS reference costs 2017/2018 updated to 2019 prices (per day) |
| Sturnem rewire elective | £1,116 | NHS reference costs 2017/2018 updated to 2019 prices (per day) |
| Transplant elective | £2,381 | NHS reference costs 2017/2018 updated to 2019 prices (per day) |
| Vascular non-elective | £519 | NHS reference costs 2017/2018 updated to 2019 prices (per day) |
| **Outpatient visits** | | |
| Ambulatory care unit | £167 | NHS reference costs 2018/2019 (per visit) |
| Anticoagulation | £37 | NHS reference costs 2018/2019 (per visit) |
| Cardiology | £139 | NHS reference costs 2018/2019 (per visit) |
| Cardiothoracic surgery | £238 | NHS reference costs 2018/2019 (per visit) |
| Clinical immunology | £286 | NHS reference costs 2018/2019 (per visit) |
| Clinical oncology | £143 | NHS reference costs 2018/2019 (per visit) |
| Clinical oncology (previously radiotherapy) | £143 | NHS reference costs 2018/2019 (per visit) |
| Clinical psychology | £199 | NHS reference costs 2018/2019 (per visit) |
| Colorectal surgery | £121 | NHS reference costs 2018/2019 (per visit) |
| Dental medicine | £138 | NHS reference costs 2018/2019 (per visit) |
| Dermatology | £113 | NHS reference costs 2018/2019 (per visit) |
| Diabetic medicine | £142 | NHS reference costs 2018/2019 (per visit) |
| Diagnostic imaging | £32 | NHS reference costs 2018/2019 (per visit) |
| Dietetics | £85 | NHS reference costs 2018/2019 (per visit) |
| Endocrinology | £161 | NHS reference costs 2018/2019 (per visit) |
| ENT | £107 | NHS reference costs 2018/2019 (per visit) |
| Gastroenterology | £141 | NHS reference costs 2018/2019 (per visit) |
| General medicine | £167 | NHS reference costs 2018/2019 (per visit) |
| General surgery | £134 | NHS reference costs 2018/2019 (per visit) |
| Gynaecology | £141 | NHS reference costs 2018/2019 (per visit) |
| Haematology | £167 | NHS reference costs 2018/2019 (per visit) |
| Hepatology | £196 | NHS reference costs 2018/2019 (per visit) |
| Hernia procedures | £219 | NHS reference costs 2018/2019 (per visit) |
| Index outpatient | £127 | NHS reference costs 2018/2019 (per visit) |
| Infectious diseases | £291 | NHS reference costs 2018/2019 (per visit) |
| Interventional radiology | £93 | NHS reference costs 2018/2019 (per visit) |
| Liaison psychiatry | £210 | NHS reference costs 2018/2019 (per visit) |
| Maxillo-facial surgery | £124 | NHS reference costs 2018/2019 (per visit) |
| Nephrology | £164 | NHS reference costs 2018/2019 (per visit) |
| Neurology | £177 | NHS reference costs 2018/2019 (per visit) |
| Oncology | £143 | NHS reference costs 2018/2019 (per visit) |
| Ophthalmology | £98 | NHS reference costs 2018/2019 (per visit) |
| Pain management | £157 | NHS reference costs 2018/2019 (per visit) |
| Phlebotomy | £4 | NHS reference costs 2018/2019 (per visit) |
| Physiotherapy | £58 | NHS reference costs 2018/2019 (per visit) |
| Plastic surgery | £107 | NHS reference costs 2018/2019 (per visit) |
| Podiatry | £54 | NHS reference costs 2018/2019 (per visit) |
| Respiratory medicine | £157 | NHS reference costs 2018/2019 (per visit) |
| Respiratory physiology | £120 | NHS reference costs 2018/2019 (per visit) |
| Respiratory sleep study | £85 | NHS reference costs 2018/2019 (per visit) |
| Rheumatology | £147 | NHS reference costs 2018/2019 (per visit) |
| Smoking cessation support | £143 | NHS reference costs 2018/2019 (per visit) |
| Stroke medicine | £197 | NHS reference costs 2018/2019 (per visit) |
| Trauma & orthopaedics | £120 | NHS reference costs 2018/2019 (per visit) |
| Urology | £108 | NHS reference costs 2018/2019 (per visit) |
| **Day cases** | | |
| Angioplasty | £1,970 | NHS reference costs 2018/2019 (per case) |
| Biopsy | £607 | NHS reference costs 2018/2019 (per case) |
| Cardiac catheterisation | £1,092 | NHS reference costs 2018/2019 (per case) |
| Cardiac | £1,052 | NHS reference costs 2018/2019 (per case) |
| Cataract | £914 | NHS reference costs 2018/2019 (per case) |
| Chemotherapy | £110 | NHS reference costs 2018/2019 (per case) |
| Colonoscopy | £608 | NHS reference costs 2018/2019 (per case) |
| Defibrillator | £2,336 | NHS reference costs 2018/2019 (per case) |
| Dermatology | £706 | NHS reference costs 2018/2019 (per case) |
| Diabetic | £744 | NHS reference costs 2018/2019 (per case) |
| Diagnostic imaging | £342 | NHS reference costs 2018/2019 (per case) |
| Dialysis | £964 | NHS reference costs 2018/2019 (per case) |
| Echocardiogram | £614 | NHS reference costs 2018/2019 (per case) |
| Endoscopy | £621 | NHS reference costs 2018/2019 (per case) |
| ENT | £423 | NHS reference costs 2018/2019 (per case) |
| Eye procedures | £401 | NHS reference costs 2018/2019 (per case) |
| Foot procedures | £1,646 | NHS reference costs 2018/2019 (per case) |
| Gastroenterology | £524 | NHS reference costs 2018/2019 (per case) |
| Hand procedure | £1,547 | NHS reference costs 2018/2019 (per case) |
| Implant defibrillator | £2,336 | NHS reference costs 2018/2019 (per case) |
| Index cost | £752 | NHS reference costs 2018/2019 (per case) |
| Kidney | £1,003 | NHS reference costs 2018/2019 (per case) |
| Multiple stent | £1,328 | NHS reference costs 2018/2019 (per case) |
| Pacemaker | £1,953 | NHS reference costs 2018/2019 (per case) |
| Respiratory | £563 | NHS reference costs 2018/2019 (per case) |
| Shoulder procedure | £2,232 | NHS reference costs 2018/2019 (per case) |
| Sigmoidoscopy | £443 | NHS reference costs 2018/2019 (per case) |
| Stent | £1,075 | NHS reference costs 2018/2019 (per case) |
| Urology | £440 | NHS reference costs 2018/2019 (per case) |
| **Accident and emergency** | | |
| No hospital admission | £144 | NHS reference costs 2018/2019 (per attendance) |
| With hospital admission | £261 | NHS reference costs 2018/2019 (per attendance) |
| **Primary, community and social care** | | |
| Anticoagulant service | £37 | NHS reference costs 2018/2019 (per visit) |
| Asthma nursing | £91 | NHS reference costs 2018/2019 (per visit) |
| Blood test | £4 | NHS reference costs 2018/2019 (per visit) |
| Breast clinic | £32 | NHS reference costs 2018/2019 (per visit) |
| Cardiac nursing | £84 | NHS reference costs 2018/2019 (per visit) |
| Care worker | £12 |  |
| Community or primary care based cardiac unit | £84 | NHS reference costs 2018/2019 (per visit) |
| Counsellor or mental health worker | £35 | PSSRU 2019 |
| Dentist | £17 | PSSRU 2019 (cost per hour used and ten minute appointment assumed) |
| Diabetic nursing | £72 | NHS reference costs 2018/2019 (per visit) |
| District nurse | £40 | NHS reference costs 2018/2019 (per visit) |
| Drug and alcohol | £91 | NHS reference costs 2018/2019 (per visit) |
| GP (at the surgery/practice) | £33 | PSSRU 2019 |
| GP (at your home) | £85 | PSSRU 2019 |
| GP (phone call) | £16 | PSSRU 2019 |
| Health visitor | £57 | NHS reference costs 2018/2019 (per visit) |
| Home care worker/home help | £12 | PSSRU 2019 (cost per hour used and assumed 30 minutes of support provided as the majority of visits lasted <30 minutes) |
| Influenza vaccination | £17 | PSSRU 2019 (for nurse administration) and BNF |
| Mental health nurse | £70 | NHS reference costs 2018/2019 (per visit) |
| Nurse (at your home) | £40 | NHS reference costs 2018/2019 (per visit) |
| Occupational therapist | £86 | NHS reference costs 2018/2019 (per visit) |
| Ophthalmology | £98 | NHS reference costs 2018/2019 (per visit) |
| Physiotherapist | £62 | NHS reference costs 2018/2019 (per visit) |
| Podiatrist | £43 | NHS reference costs 2018/2019 (per visit) |
| Practice nurse (at the surgery) | £6 | PSSRU 2019 (cost per hour) and PSSRU 2015 (average duration of contact) |
| Social worker | £45 | PSSRU 2019 |
| Stroke rehabilitation | £92 | NHS reference costs 2018/2019 (per visit) |
| Urology | £108 | NHS reference costs 2018/2019 (per visit) |
| Walk-in centre | £33 | PSSRU 2019 |
| **^1^** The NHS Reference costs 2018/2019 does not report length of stay which prevents a cost per day cannot be calculated, therefore the 2017/18 costs NHS reference costs have been used for inpatient stay costs and inflated to 2019 prices.  ^2^ If was unknown or unclear whether an admission was planned or unplanned, but it is known the admission an A&E admission occurred during the same period, then it has been costed using the non-elective inpatient costs. If there is no evidence of admission via A&E (or other info to suggest unplanned) the inpatient stay was be costed using the elective inpatient costs. If it is unclear (i.e., missing A&E data the weighted average of non-elective and elective was used).  ^3^ If the service use questionnaire indicated that an inpatient stay had been missed (i.e., participants stated an A&E visit leading to admission but no admission) the average weighted cost of a non-elective cardiac related admission was used. | | |

# Supplementary Table 2: EQ-5D domains and utility (complete case)

|  | **Usual care (n=91)** | **MCT plus usual care (n=88)** |
| --- | --- | --- |
|  | **Frequency (proportion)** | |
| **Domain** | | |
| **No problems with mobility** | | |
| Baseline | 39 (42.86%) | 31 (35.23%) |
| 4-month follow-up | 38 (41.76%) | 42 (47.73%) |
| 12-month follow-up | 35 (38.46%) | 38 (43.18%) |
| **No problems with self-care** | | |
| Baseline | 76 (83.52%) | 64 (72.73%) |
| 4-month follow-up | 70 (76.92%) | 66 (75.00%) |
| 12-month follow-up | 67 (73.63%) | 58 (65.91%) |
| **No problems with usual activity** | | |
| Baseline | 18 (19.78%) | 14 (15.91%) |
| 4-month follow-up | 30 (32.97%) | 32 (36.36%) |
| 12-month follow-up | 34 (37.36%) | 34 (38.64%) |
| **No problems with pain or discomfort** | | |
| Baseline | 23 (25.27%) | 21 (23.86%) |
| 4-month follow-up | 27 (29.67%) | 28 (31.82%) |
| 12-month follow-up | 24 (26.3%7) | 26 (29.55%) |
| **No problem with anxiety or depression** | | |
| Baseline | 18 (19.78%) | 20 (22.73%) |
| 4-month follow-up | 23 (25.27%) | 34 (38.64%) |
| 12-month follow-up | 25 (27.47%) | 33 (37.50%) |
| **Utility** | **Mean (SE)** | |
| Baseline | 0.642 (0.019) | 0.607 (0.025) |
| 4-month follow-up | 0.643 (0.024) | 0.665 (0.030) |
| 12-month follow-up | 0.642 (0.027) | 0.645 (0.029) |

# Supplementary Table 3: Costs by categories of service use (complete case)

| **Service use category** | **Usual care (n=91)** | | **MCT plus usual care (n=88)** | |
| --- | --- | --- | --- | --- |
|  | **Mean (SE)** | **95% CI** | **Mean (SE)** | **95% CI** |
| **Inpatient** | | | | |
| Pre-baseline | £4659 (£800) | £3069; £6248 | £5372 (£770) | £3842; £6901 |
| 4-month follow-up | £1105 (£539) | £34; £2177 | £566 (£206) | £157; £975 |
| 12-month follow-up | £1110 (£323) | £468; £1753 | £718 (£258) | £205; £1232 |
| **Outpatient** | | | | |
| Pre-baseline | £174 (£24) | £126; £222 | £197 (£31) | £136; £259 |
| 4-month follow-up | £154 (£20) | £114; £194 | £223 (£35) | £152; £293 |
| 12-month follow-up | £216 (£37) | £142; £289 | £310 (£51) | £208; £411 |
| **Day case** | | | | |
| Pre-baseline | £288 (£81) | £127; £449 | £99 (£32) | £36; £163 |
| 4-month follow-up | £53 (£28) | £2; £109 | £103 (£47) | £10; £197 |
| 12-month follow-up | £78 (£32) | £15; £141 | £235 (£84) | £68; £403 |
| **Accident and emergency** | | | | |
| Pre-baseline | £189 (£22) | £145; £234 | £219 (£21) | £178; £260 |
| 4-month follow-up | £55 (£14) | £26; £84 | £53 (£15) | £23; £83 |
| 12-month follow-up | £84 (£20) | £45; £123 | £81 (£20) | £41; £120 |
| **Primary, community and social care** | | | | |
| Pre-baseline | £149 (£23) | £103; £195 | £140 (£12) | £117; £163 |
| 4-month follow-up | £140 (£17) | £107; £173 | £185 (£27) | £131; £239 |
| 12-month follow-up | £239 (£39) | £162; £316 | £215 (£34) | £147; £282 |
| **Usual care and MCT** | | | | |
| MCT intervention | NA | NA | £238 (£12) | £213; £263 |
| Cardiac rehabilitation | £520 (£29) | £463; £577 | £599 (£27) | £545; £654 |
