# Supplementary material for: Cost-effectiveness of metacognitive therapy for cardiac rehabilitation participants with symptoms of anxiety and/or depression: analysis of a randomised controlled trial
Source: BMJ Open. 2024 Dec 20;14(12):e087414. doi: 10.1136/bmjopen-2024-087414 (PMC11667381; doi:10.1136/bmjopen-2024-087414)
Supplement: Supplementary file 2 [file bmjopen-2024-087414.reviewer_comments.pdf]

PEER REVIEW HISTORY

BMJ Open publishes all reviews undertaken for accepted manuscripts. Reviewers are asked to complete a checklist review form and are provided with free text boxes to elaborate on their assessment. These free text comments are reproduced below.

ARTICLE DETAILS

Title (Provisional)

Cost-effectiveness of metacognitive therapy for cardiac rehabilitation participants with symptoms of anxiety and/or depression: analysis of a randomised controlled trial

Authors

Shields, Gemma E; Camacho , Elizabeth; Davies, Linda M; Doherty, Patrick Joseph; Reeves, David; Capobianco, Lora; Heagerty, Anthony; Heal, Calvin; Buck, Deborah; Wells, Adrian

---

VERSION 1 - REVIEW

**Reviewer**	**1**
**Name**	**Senanayake, Sameera**
**Affiliation**	**Queensland University of Technology**
**Date**	**30-Apr-2024**
**COI**	**None**

Thank you for giving me the opportunity to review this manuscript. I believe that the manuscript is well-written and offers valuable insights. My only suggestion is to include a paragraph in the discussion section addressing the uncertainty observed in the results. This paragraph could cover the following points:

• Sources of uncertainty: This would involve identifying potential sources of uncertainty such as data variability, small sample sizes, missing data, or limitations in measurement tools and techniques.

• It would be beneficial to discuss any methodological limitations that could contribute to the observed uncertainty.

• It is crucial to consider whether the findings are robust enough to guide clinical actions or if further research is needed to solidify these conclusions.

• Suggesting strategies or methods that could be employed in future studies to reduce uncertainty and improve the reliability of the findings.

Reviewer 2

Name Pepera, Garyfallia

Affiliation University of Thessaly School of Health Sciences, Department of Physiotherapy, Clinical Exercise Physiology and Rehabilitation Laboratory

Date 19-Jun-2024

COI None

Dear authors,

This is a really interesting topic, however, the comments below need to be answered before the manuscript can be considered for acceptance.

Introduction

Make clearer the aim of this study. What is the main aim, secondary etc.

Add previous scientific knowledge on this area

Methods

Should be improved and add more details

Inclusion criteria should be clearer: what about age, population. Inclusion and exclusion criteria.

Say more about exercise program, applications, supervised, length. Did you include tele-rehab?

Discussion

Line 36-46: 'Prior reviews...' Please expand and say why there are all these differences between studies.

'During the COVID pandemic..." 'Research is needed to

compare the effectiveness and cost-effectiveness of clinic-delivered MCT versus home-based MCT in CR, and this is likely to need to consider patient preferences and how this will affect uptake/attendance.'

There is evidence (suggested REFs)

What other knowledge exists in cost-effectiveness for telerehab/remote CR?

What are the clinical findings/clinical importance?

What about study strengths and limitations? Barriers for CR?

Suggested:

-Cardiac telerehabilitation in a middle-income country: analysis of adherence, effectiveness and cost through a randomized clinical trial [published online ahead of print, 2022 May 30]. Eur J Phys Rehabil Med. 2022;10.23736/S1973-9087.22.07340-3. doi:10.23736/S1973-9087.22.07340-3

-The cost-effectiveness of exercise-based cardiac telerehabilitation intervention: a systematic review. European Journal of Physical and Rehabilitation Medicine 59 (2), 248. doi: 10.23736/S1973-9087.23.07773-0

- Safety and long-term outcomes of remote cardiac rehabilitation in coronary heart disease patients: A systematic review

---

VERSION 1 - AUTHOR RESPONSE

**Reviewer 1**	
Thank you for giving me the opportunity to review this manuscript. I believe that the manuscript is well-written and offers valuable insights.	Thank you for the time taken to review the manuscript, we appreciate your feedback.
My only suggestion is to include a paragraph in the discussion section addressing the uncertainty observed in the results.<br><br>This paragraph could cover the following points:<br><br>• Sources of uncertainty: This would involve identifying potential sources of uncertainty such as data variability, small sample sizes, missing data, or limitations in measurement tools and techniques.<br>• It would be beneficial to discuss any methodological limitations that could contribute to the observed uncertainty.<br>• It is crucial to consider whether the findings are robust enough to guide clinical actions or if further research is needed to solidify these conclusions.<br>• Suggesting strategies or methods that could be employed in future studies to reduce uncertainty and improve the reliability of the findings.	We have expanded the text in the discussion to better highlight how we address uncertainty. For example, we added "A key limitation of the economic evaluation specifically relates to uncertainty." The text following this sentence discusses missing data, the sample size, variability, service use data collection methods and how these could be improved in future strategies. We have explicitly addressed the point around clinical actions: "Whilst there are some favourable results, without more robust data, it is challenging to make conclusions around whether the roll-out of MCT nationally would be cost-effective." This also relates to the BEACONs project which is collecting further evidence and may answer this more clearly (this study is referenced in the discussion).
**Reviewer 2**	
This is a really interesting topic, however, the comments below need to be answered before the manuscript can be considered for acceptance.	Thank you for the time taken to review the manuscript, we appreciate that your points focus on the provision of further information and expanded discussion and have addressed these below.

Introduction<br>Make clearer the aim of this study. What is the main aim, secondary etc.	The aim has been highlighted more clearly at the end of the introduction and now reads: "The aim of this study was to assess whether the addition of MCT to CR for patients with elevated symptoms of anxiety and/or depression is potentially cost-effective, versus usual care. "
Introduction<br>Add previous scientific knowledge on this area	The introduction has been split up further to highlight the current lack of knowledge in this area and the prior knowledge is now a separate paragraph: "To date there have been no cost-effectiveness studies of MCT and a review of cost-effectiveness studies in CR identified only two studies evaluating other psychological support in CR, with mixed results and limited generalisability to UK practice [10]. "
Methods<br>Should be improved and add more details<br>Inclusion criteria should be clearer: what about age, population. Inclusion and exclusion criteria.	We reported the trial details in brief due to the word count restrictions. We include references to other study papers which report these topics with much more detail: "Full details of the trial methods and results, as well as wider work from the funded programme of research, are available elsewhere [16, 17, 21, 22, 25]."<br><br>The inclusion and exclusion criteria have been added under "design, setting and participants". Participant characteristics (e.g., age) are described in Table 2.<br><br>Note the paper meets the reporting requirements of the CHEERS2 checklist for economic evaluations.
Methods<br>Say more about exercise program, applications, supervised, length. Did you include tele-rehab?	Exercise programs were included as part of 'usual care' which was offered to participants in both treatment groups. The content of usual care was not mandated as part of the study therefore we cannot say more about this. The focus of this study is on the delivery of metacognitive therapy which was provided in addition to usual care. Whilst all participants will have been offered treatment as usual (which includes exercise programmes), the exact design and delivery of this will vary by location. It has been clarified in the paper that this may be home and/or centre-based. Tele-rehab may have been a component of usual care, again this will differ by locality. This is noted in the limitations in relation to generalisability and has been expanded: Results may not be generalisable to other settings; cardiac services, including the type of CR

	offered (e.g. face-to-face or home-based) and exact design and delivery of components (e.g., exercise components) and populations vary by area [41, 42].”<br><br>Regarding metacognitive therapy specifically, this was delivered on-site. However, the discussion does include a paragraph on home-based care which references a separate study exploring this option.
Discussion<br>Line 36-46: 'Prior reviews...' Please expand and say why there are all these differences between studies.	This text is expanded after this sentence and makes reference to the original studies: “Two existing studies focus on psychological therapy in CR; one study of a home-based cognitive–behavioural therapy (CBT) found it to be cost-effective in the majority of cases versus usual care (67%), and another focused on learning and coping education strategies which was cost-effective only 29% of the time [38, 39].”
Discussion<br>'During the COVID pandemic..." 'Research is needed to compare the effectiveness and cost-effectiveness of clinic-delivered MCT versus home-based MCT in CR, and this is likely to need to consider patient preferences and how this will affect uptake/attendance.'<br>There is evidence (suggested REFs)<br>Suggested:<br>-Cardiac telerehabilitation in a middle-income country: analysis of adherence, effectiveness and cost through a randomized clinical trial [published online ahead of print, 2022 May 30]. Eur J Phys Rehabil Med. 2022;10.23736/S1973-9087.22.07340-3. doi:10.23736/S1973-9087.22.07340-3<br>-The cost-effectiveness of exercise-based cardiac telerehabilitation intervention: a systematic review. European Journal of Physical and Rehabilitation Medicine 59 (2), 248. doi: 10.23736/S1973-9087.23.07773-0<br>- Safety and long-term outcomes of remote cardiac rehabilitation in coronary heart disease patients: A systematic review	This text is focused specifically on the availability of cost-effectiveness evidence for home-based MCT in CR (not home-based CR more broadly, for which we know there is evidence). The suggested references have been added in response to the comment below.
Discussion<br>What other knowledge exists in cost-effectiveness for telerehab/remote CR?	There is a paragraph in the discussion focused on home-based options, a sentence has been added which summarises the cost-effectiveness evidence of home-based CR, alongside the evidence for home-based MCT: “[21, 22]. Home-based MCT has been shown to be effective in reducing symptoms of anxiety and depression in the population receiving CR [45].

	Furthermore, home-based CR options have been demonstrated to be cost-effective [46].”
Discussion<br>What are the clinical findings/clinical importance?	While the focus of this paper is cost-effectiveness evidence, this has been added/addressed in the discussion: “Whilst there are some favourable results, without more robust data, more robust data is needed to make stronger conclusions around cost-effectiveness. It should be noted that the clinical findings for Group MCT are positive; with more moderate to large reductions in mental health symptoms (e.g., anxiety and depression) when compared to CR alone [17].” For interested readers the clinical effectiveness paper is referenced, however, all results cannot be provided here due to word count limitations.
Discussion<br>What about study strengths and limitations?	The limitations are included throughout the discussion. In response to the editor the key strengths and limitations have been revised at the start of the paper (with a focus on methods). A statement summarising the strengths of the economic evaluation has been added: ”Key strengths of the economic evaluation include the prospective collection of economic data alongside a robust randomized-controlled trial, comprehensive investigation into uncertainty and a range of sensitivity analysis to explore the impact of assumptions related to methods/design.” The strengths and limitations of the overall study and clinical evaluation are presented in full in the separate paper reporting those findings.
Discussion<br>Barriers for CR?	This was noted in the Discussion and has now been expanded: “Important inequalities in uptake exist in CR, with lower uptake in more deprived areas, for people from minority ethnic groups, for single and older people [40, 41]. Furthermore, practical barriers exist, such as financial costs, travel time and the need to take time off work/other commitments to attend [42].[…]Furthermore, research into how MCT could avoid or overcome the current barriers to CR attendance would be beneficial.”

VERSION 2 - REVIEW

Reviewer 2

Name **Pepera, Garyfallia**

Affiliation **University of Thessaly School of Health Sciences, Department of Physiotherapy, Clinical Exercise Physiology and Rehabilitation Laboratory**

Date **17-Nov-2024**

COI

Most of the comments were addressed. I would endorse publication.
